# Discussions before the Mississippi Valley Dental Association

**Published:** 1875-05

**Authors:** 


### Proceedings of Societies.

### DISCUSSIONS BEFORE THE MISSISSIPPI VALLEY DENTAL ASSOCIATION.

WEDNESDAY MORNING, MARCH 3d, 1875.

Dr. H. A. Smith read an essay written by L. G. Noel, of Nashville, (that gentleman being absent.) Subject; "The prevention and treatment of caries upon the proximate surfaces of teeth." After the reading of the paper the subject was announced as open for discussion.

Dr. Keeley said that he approved in the main, of all that had been set forth in the essay. He does not always resort to extracting to relieve a crowded denture, in the case of a young patient; since the expansion of the arch will in time, frequently correct such irregularity. Does not approve of Dr. Arthur's method of anticipating decay by cutting spaces between sound teeth. Sometimes employs the method, where the proximate surfaces are already slightly or even considerably decayed, being particular to thoroughly polish the exposed dentine afterward.

Dr. Hunt expressed his approval of Dr. Keeley's views. Said that he practices cutting away superficial decay to arrest the disease more than he formerly did. Where it is practicable however he advocates contour fillings.

Dr. Rawls asked for an expression from the members as to the expediency of cutting away the proximal snrface of the temporary molar as a means of preventing decay of the permanent molars.

Dr. Taft: No invariable rule of practice can be prescribed· Sometimes it is best to cut away to prevent decay or to arrest it after it has occurred; in another case you may find it necessary to extract to accomplish the same object. Again, it may be inexpedient to extract, where by so doing you would only perpetuate an irregularity which might have been corrected by the use of proper appliances.

The liability of the teeth to decay must enter into the consideration. In general I make it rule th extract one or more teeth in cases of irregularity where the indications are that nature will then remedy the defect. Much judgment in this matter, and a nice discrimination are required.

Dr. Morgan: There is a class of teeth which have not been mentioned, which I should remove to gain room. If the six year molars are decayed and there is a good prospect of saving the neighboring teeth by sacrificing them, I frequently do it. First, because it gives a better opportunity for the regular development of the dens-sapientia and offers facilities to the bicuspids to separate a little; and in the second place because, if these teeth were filled instead of extracting them the probability is that it would be necessary to extract them after a few years. And the final result would not be as satisfactory as if they had been extracted in the first place.

Dr. Taft: Do you pursue that course where the teeth are not specially crowded?

Dr. Morgan: Yes, sir. I should remove them where they were merely in contact with their neighbors. If there was a crowded condition, that fact would operate as an additional eason for extracting them.

Dr. Taft: I think you should be goverened by the extent of the decay and the predisposition to decay. We should consider the peculiarties of individual cases. You might do for one patient what you should not do for another where the teeth are in apparently the same condition. Unless there is a marked tendency to decay it is not by any means, in my judgment, always best to extract these teeth. Frequently they can be saved keeping the teeth thoroughly clean especially on the proximate surfaces.

Dr. Watt: In my intercourse with my professional brethern I find that nearly all are agreed that if the fluids of the mouth have an alkaline reaction the danger of decay is not imminent. Some members of the profession in the East are beginning to think that the alkalies induce decay. They act upon the organic portion of the tooth. When decomposition results in the formation of ammonia, caries is more likely to result than if the fluids of the mouth were slightly acid. White decay, such as is often found on proximal surfaces and at the necks of teeth, results from the action of nitric acid. The formation of nitric acid results from the presence in the mouth of ammonia. It is hence important that we should take means to discover whether an alkaline reaction is the result of the presence of ammonia in the mouth. The condition is often found in the case of young patients and indicates that the patient has abstained from the use of acid fruits, etc. Prescribe for him acid fruits to be eaten after dinner, or lemonade, pickels, etc.

Dr. Smith: I presume we all know that a space between teeth renders them less liable to decay, whether we have an acid or an alkaline reaction. There may be a predisposing cause of decay in the pressure of the approximal surfaces of the teeth against each other, thus causing a lowering of vitality of the tooth and resulting in a withdrawal of the earthy constituents, thus weakening the tooth or teeth. This theory is earnestly advocated by Prof. Chase, of St. Louis. It may be the extraction of teeth to relieve this crowded condition, is correct practice.

In answer to Dr. Rawls question I will state that I have frequently, since I read Dr. Arthur's book, separated the deciduous teeth from the six year molars. I do not mean to offer an unqualified indorsement of Dr. Arthur's method. He bases his theory upon the assumed fact that the dentine has the greater resisting power to decay; do not like to remove the enamel.

Dr. Rawls would hesitate to separate the deciduous tooth from the six year molar for the reason that he thinks the contact of the teeth necessary for the proper development of the jaws of the child. I think we might remove every deciduous tooth without effecting the conformation of the jaws. The lower base of the jaw is a part which has no relation to the alveolar process at all, so far as this question is concerned. The extraction of a deciduous tooth does not necessarily effect a change in the shape of the jaws.

Dr. Rawls: I do not think there can be a perfect development of the jaw unless the teeth are present. The pernicious effects of extracting the temporary teeth may not always be apparent, and perhaps are not often traced to their true cause. As to separating the teeth, I do not approve of cutting away the milk molar, particularly for the reason that the teeth thus separated are left with the cervical walls in contact and irritation is the result. If you make a deeper separation you have not prevented contact of the surface and sometimes you have a tipping forward of a permanent tooth consequent upon this operation. How many young persons of the age of fourteen or fifteen years do we find whose molar teeth antagonize perfectly.

Dr. Canine: There is an anatomical reason why it is impossible for the six year molar to tip forward as a result of cutting away the temporary tooth. The roots of the latter we find in almost every case are larger at the termination of the enamel: they are wider from before backward than the crown. So that the removal of the surfaces of enamel in contact, does not in fact relieve the pressure and support of the teeth unless the the cut is made quite deep. It will be found almost invaribly that the permanent bicuspid crown presents itself between the roots of the temporary teeth and prevents their being pressed together. It is all important to keep the spaces clean after the separation has been made.

I think we find in the majority of cases that the mesial surfaces of the molars are decayed and in my opinion it might have been prevented by free separation.

Dr. Rawls: The theory based on the anatomical structure of the teeth will not I think, obtain in the majority of cases, because physiological conditions are so frequently unfavorable. I would like to have the gentleman examine carefully and see how frequently the second molar assumes a position considerably below the crown surface of the first molar, as a consequence of this tipping forward.

Dr. Morgan: I think if we will take the trouble to observe in most cases we will find that at six years of age none of the incisors are in contact. There is not only *not* a wedged condition but there is not even contact. At that age there is an enlargement of the front portion of the maxilla causing a separation of the teeth to give room for the eruption of the permanent set.

It has been ascertained that the *enlargement* of the jaw strictly speaking is confined to the portion back of the molar tooth, forward of the six year molar the jaw *elongates.* It will be found by actuaal measurement that the two temporary molars and canine teeth occupy just about as much space as the two permanent bicuspids and the canine tooth. So the idea of their being wedged apart is incorrect. I only advocated positively the removal of every single dead six year molar up to about fourteen years of age; in order to preserve the contiguous teeth. We see these teeth time and again in the mouths of patients of eight and ten years, in a condition which precludes the possibility of saving them by ordinary means. The sooner that fact is appreciated the better. For the prevention of decay between teeth where I think it is otherwise inevitable, I frequently separate all the teeth. I do the same thing to remove decay where it has already commenced I consider it sound practice to seperate these teeth and polish them carefully where there is a predisposition to decay.

Dr. Watt: I will state that while caries is ordinarily due to the action of acids, these acids must be in a nascent condition. In a passive condition they do not operate.

Dr. Berry: Thirty years ago the practice of separating teeth was very common, even in cases where now we would insert fillings. The success of that mode of treatment in cases which I have seen is very apparent. I have seen the teeth of patients in good state of preservation forty years after the operation. There was no evidence of a tendency to decay or disintegration of the dentine; on the contrary it had become hard and dense, The person had always taken good care of the teeth. I have seen another case of twenty-seven year's standing, all the incisors had been freely separated to remove decay, and were in perfect condition. The patient used a brush with but one row of bristles—using it after each meal.

Dr. Richardson: I think nature originally designed that there should be room for the full complement of teeth, at the proper time. I do not consider teeth to be in a crowded condition when they are merely in contact. I think that nature designed that they should be in contact; each tooth serving as a "keystone" to preserve the shape of the arch, and to aid in a proper and symmetrical development of the jaws in their relations to each other. The removal of a tooth under such conditions, may result in serious injury. I should hardly feel justified in extracting any tooth for the mere purpose of preventing decay or for the treatment of decay. If there was mal-arrangement, that I would consider a crowded condition. In that condition it is often advisable to extract.

Dr. Taft: Dr. Rawls' remarks about the tendency of the second molar to tip forward after the removal of the first molar, is worthy of consideration. I have not found in my practice that, as Dr. Morgan has stated, when the first permanent molar has been removed the germ of the second molar would start up immediately and take the place of the first molar. I find oftentimes however that the second molar tips forward until its anterior cusps rest against the posterior surface of the second bicuspid. Sometimes the bicuspid moves back slightly and the second molar moves forward so that we have apparently the phenomenon which he describes. These cases are exceptions.

Again, I think Dr. Morgan is in error in stating that the enamel on the approximate surfaces of the bicuspids is relatively far less perfect than elsewhere on the crowns. This is a vulnerable point it is true.

I do not believe that one tooth ever presses against another so as to cause decay. Simple contact of the teeth does not necessarily indicate decay.

Dr. Morgan: I am not skilled in the use of the microscope but my observation has led me to believe that there is a defect of organization in the enamel of the posterior surfaces of bicuspids, especially the superior. It is thinner than elsewhere. On the lingual and buccal surfaces you will find it is thick.

Dr. Jas. Taylor: It has been generally assumed by the profession during the past thirty or forty years, that the positions of the six year molars relatively change after their perfect eruption. This theory however has been disproved by Dr. Bell who experimented with reference to the question. He ascertained that the spaces of the permanent teeth, between the anterior molars and the six year molars was 3-20 larger than that occupied by the deciduous teeth, 2-20 of this increase of space was obtained between bicuspid teeth and 1-20 by the diminished size of the bicuspids as compared with the deciduous molars. We all know that the six anterior permanent teeth are larger than the temporary teeth, I think that it will be found upon examination that as dentition progresses and the permanent teeth take their position the deciduous organs do move slightly. I do not think the deciduous teeth are often crowded. We do find however that the permanent teeth as they are erupted, point inward nine times out of ten, and each permanent tooth fills a space 1-3 larger than the space occupied by the deciduous tooth. I think by a process of accretion we have a slight expansion of the arch to accommodate the new set.

As to the anterior molars I think there is a time when they can not be extracted without affecting injuriously the position of the other teeth. They should be extracted early in life to avoid the trouble of the posterior tooth tipping forward. Then you may expect later, a well developed, perfectly organized dens sapientia to take the place of the poorly organized molar. With reference to the structure of enamel, I do think we find cases where the enamel is imperfect in its structure. Sometimes the defect of organization is due to the constitutional disability. We know that enamel is formed before the tooth is erupted. How then can any change take place in the enamel after it is erupted? The question then occurs is the tooth germ subjected to pressure which prevents the deposition of enamel? I think not. The tooth is embedded in a capsule and the enamel organ is interposed But, again, as to the extraction of the six year molar, as my friend Dr. Morgan has said, "always extract these teeth when devitalised." I extract them at seven or eight years. I want them taken out before the second molar begins to make its appearance. The sooner the better. If you extirpate the nerve in the tooth and attempt to fill the roots you are almost sure to have trouble owing to the large size of the foramina.

AFTERNOON SESSION.

The third subject was announced for discussion. "Irregularities of the teeth; description of cases; method of treat. ment."

Dr. Watt said that in correcting an irregularity when it becomes necessary to use rubbers or ligatures to move teeth into positson, there should be periods of rest, in order to avoid severe inflammation of the parts, and to favor the adaptation of the investing tissues to the tooth in its changed position. His objection to rubber ligatures is, there is danger of their doing irreparable injury by slipping up under the gum and by their persistent drawing exciting severe periostitis· He uses wedges of wood, cotton or paper.

He can wedge the strongest teeth with these agents. Would not drill holes in the teeth to insert screws for the purpose of attaching ligatures. Uses bands and clasps and accomplishes the object quite as readily. It is not neccessary, if the tooth is moved by slow degrees allowing periods for rest, to hold it in position nearly so long as is usually done. To keep the teeth apart, he sometimes uses a bit of hard sub stance as a piece of Scotch stone, with a band of rubber so applied as merely to retain it and the teeth in position. Dr. Keely has suggested the use of cotton threads but he does not like them because the tension is unequal, a condition of absolute rest is the great desideratum after the tooth has reached its proper position.

Dr. Osmond said he has drilled holes in the teeth and then passing a thread around them, saturating it with sanderac varnish. He has tried many other methods and finds this the simplest and best.

Dr. Taft related a case which he had seen under treatment at the hands of Dr. Dwinelle. The points of the cuspids were just at the margin of the gums. He expanded the arch and then drew the teeth down to their proper position. with ligatures. He regarded the successful accomplishment of this as remarkable, considering that the teeth were not fully erupted. He saw the casts of the case at several stages of progression. Could not state just how long the treatment was in progress. The operation was a perfect success.

The case was a novelty to him. It showed a remarkable adaption of the parts to movements of the teeth.

Dr. Taft mentioned a case of a lady who had a staple inserted in a tooth for the convenient attachment of a ligature The tooth decayed so as to allow the staple to drop out. He suggested that where some such appliance must be used the small screws are to be prefered.

Dr. Smith: The case related by Dr. Taft, calls to my mind a case that was presented in my office the other day. The patient was nearly thirteen years old, and the cuspids were just appearing. They were entirely outside of the arch. The superior maxillary was exceedingly small. What was most remarkable, the teeth were in close contact. You would have supposed, from the appearance, that the arch was complete. It is probable that no material improvement could be made even if the arch were expanded. The question arose whether I would be justified in expanding to the annoyance of the patient, or whether it was not better to extract the cuspids. I consulted a neighboring dentist, and he also advised the removal of the cuspids.

In regard to extracting teeth to correct irregularities, I think it is sometimes advisable. It is often resorted to I think when the operator wishes to save himself trouble.

Dr. Keely: I think you can accomplish the object by removing the first bicuspid.

Dr. Osmond described a case where the cuspids projected from the gum almost at right angles. He extracted them and then dressed off the inner cusp of the bicuspid to give it the semblance of a cuspid: The sensitiveness of the exposed dentine he obtunded by applying the nitrate of silver.

Dr. Keely: I would never remove a cuspid in such a case. Would rather extract the bicuspid, and leave the cuspid to take care of itself. The pressure of the lip will bring the cuspid into position. By extracting the cuspids you mar the expression of the face.

Dr. Canine: I was struck with the remarks Dr. Watt made about the importance of retaining the teeth in a manner to secure absolute rest when you have once moved them into position. I heartily approve of the suggestion. My aim always is to save the patient all unnecessary suffering in correcting an irregularity. I think it unnecessary to subject the patient to severe suffering ordinarily. I usually employ plates to accomplish the object. I allow the points or the plate to extend between the teeth as far as possible. When the plate has expended its force I pinch down those points and direct them against the lingual surfaces of the teeth. This operation I repeat if necessary. In a case like that cited by Dr. Smith, I say extract the first bicuspid invariably, unless the patient is over fifteen or sixteen years old. It might not be best in the case of a patient twenty-five years old or older. I do not call to mind a single case in which the canine tooth failed to assume its proper position after I had extracted the bicuspid. The canine teeth I consider to be the keystone in the arch.

To extract them often causes a permanent disfiguration.

In deciding what teeth to extract in correcting irregularities or in deciding whether or not to extract at all, I am guided by various considerations.. I examine the occlusion in the general projection and fulness of the mouth, and other matters which I can not now specify.

Dr. Jay related a case of a boy nine years old. The central incisors were large. The canines occupied the place of the laterals, and were close to the centrals. The bicuspids were in position. The laterals appeared directly behind the centrals. The centrals and cuspids closed over the edges of the lower teeth. The laterals were long and impeded the patient's speech. What course should be pursued?

Dr. Canine: I exhibited an impression of a mouth on which I operated at the Indiana State Association two years ago. The laterals stood apart about one-fourth or one-eighth of an inch. The canines and centrals almost touched. Corrected that irregularity in forty days. She declared she had suffered no pain.

Dr. Smith: I would have the treatment of irregularities made a specialty. The average professional man should have nothing to do with such cases. The public must be taught to appreciate the value of such operations. How many practitioners have the nerve to tell a patient what would be regarded as an adequate compensation for the accomplishment of such results as are expected? [Subject passed.]

The Kentucky State Dental Association will hold its next annual meeting in the city of Lexington, on Tuesday, June 1st, prox. We hope the profession of Kentucky will be fully represented at that meeting, and as many visiting members from outside the state as can make it convenient will find it pleasant and profitable to be there; and all who go will find a most hearty welcome. A good programme is provided for the meeting.

